# Assessing brand switching level and behaviour of growing-up milk products in Java: A structural equation modeling and multigroup analysis

**DOI:** 10.1016/j.heliyon.2023.e15969

**Published:** 2023-05-19

**Authors:** Sunardi, Jangkung Handoyo Mulyo, Irham, Jamhari

**Affiliations:** a Agribusiness Management, Faculty of Agriculture, Universitas Gadjah Mada (UGM), Indonesia; b Department of Agricultural Socio-Economics, Faculty of Agriculture, Universitas Gadjah Mada (UGM), Indonesia

**Keywords:** Brand switching level, Brand switching behavior, Growing-up milk products, Structural equation modeling, Multi Group Analysis

## Abstract

The biggest consumers of dairy products for children aged 1–3 years, which are referred to as growing-up milk (GUM), come from the middle and lower socioeconomic classes. More than 90% of Indonesians belong to this segment. In 2020, the proportion of the population living in rural and urban areas will be almost equal, namely 43.3% and 56.7%, respectively. Understanding brand switching behavior is essential to enabling GUM manufacturers to stay in business and thrive by retaining loyal customers. The aims of this study are (i) to assess the level of brand switching; (ii) to examine the determinant factors of brand switching behavior; and (iii) to compare the brand switching behavior of GUM consumers in rural and urban areas of middle and lower socioeconomic classes in Java. The research was conducted in 4 sub-districts in 2 provinces (East Java and D.I. Yogyakarta) using a guided interview method with a questionnaire. Research respondents were 419 consumers of GUM, and they were selected using the purposive sampling method. Data analysis used partial least squares - structural equation modeling (PLS-SEM) and multigroup analysis (MGA). The study found that the level of brand switching among GUM consumers in Java is 57%, which is considered high. The most important factor that influences the brand switching behavior of GUM consumers in Java's middle and lower socioeconomic classes is bad prior experiences, followed by variety seeking, bad product attributes, and customer dissatisfaction. A defective product is the most reflective indicator of a bad prior experience. There is no difference in brand switching behavior between rural and urban consumers in Java from the middle to lower socioeconomic classes. Therefore, GUM manufacturers are allowed to adopt the same marketing strategy to increase efficiency.

## 1 Introduction

The shifting of a customer's purchases from one brand to another for specified reasons is known as brand switching. It can also be perceived as the vulnerability of a consumer to switch to another brand [1]. When consumers switch brands, short-term sales revenues diminish and long-term market share declines [[2], [3], [4]]. Due to the importance of sales revenue, it is thus positioned at the top line of the income statement [5].

In Indonesia, growing-up milk (GUM) products account for more than three-quarters of volume sales and more than two-thirds of sales revenue in the baby food market [6], which totals US$18.56 billion or equal to IDR 269.12 trillion [7]. It is obvious that a one percent decline in the company's market share may result in a loss of IDR 2.69 trillion, which is a significant sum of money.

GUM products are sold as milk that is formulated with higher nutrition content, like proteins, vitamins, and minerals, and are designed for toddlers aged one to three years. GUM products are able to take care of insufficient nutritional intake during the growing period [8].

Brand switching is a very dynamic phenomenon that is always evolving. These changes depend on how each company's marketing plan attempts to win the extremely tough competition to keep or grow sales. To preserve revenue, a corporation has to prevent brand switching. When a lot of customers switch brands, it may be the main reason why the business fails.

Brand switching of GUM products happens frequently enough to be noticeable. In the last five years, a GUM manufacturer has lost more than five percent of its users owing to brand switching, as seen by a drop in their market share, according to market statistics. In contrast, other businesses obtained almost 10% of the market share.

The occurrence of brand switching may be tracked by observing the shifting of the winning brands in the top brand index, which is maintained in Indonesia by various consumer research organizations such as Frontier Group and published in a marketing or business magazine [9]. Alterations in the market share of products within the same category might also indicate brand switching. Nielsen and other reputable global consumer research and marketing businesses offer market share information.

Therefore, the level of product brand switching as opposed to brand loyalty must be quantified. How frequently a customer purchases the same brand is a sign of their loyalty. This is called the behavioral perspective (stochastic) [10]. Customer loyalty is proportional to the share of total purchases attributable to the preferred brand. This metric is based on the proportion of purchases with hard core criteria (the single most frequently purchased brand). Consumers are considered loyal if they purchase over 75% of their preferred brand [10].

Stopping customers from switching to other brands is a key business challenge that must be met if the company wants to keep its loyal customer base. Yet, acquiring new customers is going to be significantly more expensive than retaining existing ones. Therefore, apprehending the brand and consumer demeanor is crucial, due to product innovation process requires customer needs and insight [11]. Referring to Refs. [12,13], the cost of maintaining current loyal consumers is less than the cost of acquiring new ones. It can even be five times more expensive, depending on the kind of study and industry. Companies need to focus on retaining existing customers rather than putting too much effort and resources into attracting new ones. Switching eventually erodes profitability since retaining existing customers is far less costly than acquiring new ones [14]. In order to save expenses and foster long-term consumer connections, it is crucial to analyze the variables influencing brand switching. Once these variables have been recognized, businesses may build brand management tactics that dissuade consumers from switching brands [15,16].

Numerous studies have examined the variables that influence brand switching behavior. The variable of bad previous experiences stimulates switching of product brands [[17], [18], [19]]. Inadequate product attributes, such as packaging, price, quality, and service, induce brand switching [[19], [20], [21], [22], [23], [24]]. Consumer dissatisfaction is a circumstance in which consumers' expectations are not met or exceeded by marketers' performance. Consumers frequently want diversity and are driven to switch brands if they are dissatisfied with a prior product. Brand switching is associated with consumer dissatisfaction [2,17,[25], [26], [27], [28], [29], [30]].

Variety seeking is the quest for new brand variants, or variety. It is a spontaneous purchase made by customers with the intention of trying different brands of a product. In this scenario, buyers frequently switch brands to seek diversity [19,26,28,[31], [32], [33], [34]]. There is no one model that adequately explains why consumers switch brands in various industries and for various products. Typically, each study employs a distinct combination of independent variables to explain the behavior of brand switching, depending on the business or product being examined [35].

More than 90% of Indonesia's population belongs to the consumer category of the middle and lower socioeconomic classes, where the maximum monthly household expenditure is IDR 5.0 million [36]. Consequently, the main buyers of GUM products are members of these social classes. About 56.7% of the population of Indonesia lives in urban regions, while 43.3% lives in rural areas [37].

Java is one of the largest and most prominent islands in Indonesia. Java Island, which covers around 6.75% of Indonesia's total land area, is home to 151.6 million people, or 56.10% of the country's population. More than 58% of the economic structure of Indonesia will be made up of provinces on the island of Java [38].

The customers in rural and urban areas have distinctive brand selection patterns. In general, Indonesian consumers have trust in conventional media and tend to believe marketing messages, advertisements, and salespeople's recommendations, which makes them more likely to try new products from a particular brand [36]. The brand itself has the greatest influence on developing consumer loyalty in urban areas [39]. According to Ref. [40], compared to rural consumers, urban consumers have a higher tendency to select the most popular brands.

This study describes the level of brand switching that happens and the variables that have the greatest impact on the probability of brand switching, particularly for rural and urban customers. The research came up with a quantification of brand switching levels based on a behavioral perspective (stochastic). Implementing partial least square - structural equation modeling, the research provided current evidence by explicating causal linkages between bad prior experiences, bad product attributes, and consumer dissatisfaction, including variety seeking with regard to brand switching. Using multigroup analysis, the research compared rural and urban consumers' tendency to switch brands.

This research gives us a better understanding of how frequently and why people switch GUM product brands. This can help us come up with the right marketing strategy to maintain the company's GUM product business and prevent a decline in sales. The purposes of this study are (i) to evaluate the rate or level of switching of brands; (ii) to investigate the determinant factor of switching brand behavior, and (iii) to compare the brand switching behavior of rural and urban GUM consumers from the middle class and lower class socioeconomic groups in Java.

## 2 Review of literature

### 2.1 Literature review of brand switching

The decision to switch from one brand to another is a dynamic and multifaceted process involving several behavioral factors [41]. A consumer's reluctance to switch brands as a result of positive prior brand-purchasing experiences [19]. Without adequate product attributes, consumers may switch brands [42]. The need for variety and consumer dissatisfaction were strongly associated with brand switching [26].

Fast moving consumer goods (FMCG) are typically low-priced products that sell rapidly. Additionally known as consumer packaged goods. Particularly, these FMCGs have low profit margins and high volume of sales [43]. Dairy products, notably GUM, are a significant portion of FMCG's business. Due to the high turnover rate of fast-moving consumer goods, the market is not only enormously large but also extremely competitive. Procter & Gamble, Nestlé, and Coca-Cola are three of the major manufacturers of fast-moving consumer goods in the world. Nestlé, located in Switzerland, operates approximately 2000 brands. In the field of fast-moving consumer products, competition for market share is intense [43].

FMCG products are often exchanged, resulting in great volume and low price owing to repeated purchases, and they are the first to be removed from the supermarket or hypermarket shelves. The food products market represents 43% of the entire market share [44]. Therefore, brand loyalty is tough to build and retain in the FMCG industry, and customers are likely to switch brands. Methods such as repurchase propensity provide a relatively precise evaluation of brand loyalty among FMCG customers [45].

According to Ref. [46], consumers' prior product experiences are a significant aspect of their decision to purchase or switch brands. Other previous studies by Refs. [47,48] revealed that when previous purchases produced negative results, consumers switched to other brands of products. Furthermore [[17], [18], [19]], discovered that bad previous purchasing experiences will drive consumers to switch brands. However, according to Ref. [17], Westerners and Easterners switch brands differently, when individuals have had a negative experience purchasing a product in the past. Each culture's approach to switching brands after a previous negative experience will differ.

In FMCG, the influence of good consumer experiences on consumers' long-term memories results in good brand attitudes and, ultimately, brand loyalty [49], the consumer will not thus switch brands. Customer brand loyalty is formed by the accumulation of favorable or good experiences over the past [50]. Therefore, consumers will almost certainly switch brands if they had a bad experience with their previous purchase. Customers that have a good experience with a certain brand will become brand loyalists [51]. When consumers choose a product brand, the product experience is crucial [52,53]. However, according to Ref. [17], the approach of each culture to switching brands after a prior negative experience will vary. Specifically, this study aims to examine if there are behavioral differences between rural and urban consumers when it comes to switching GUM product brands.

In relation to customer preferences, product attributes have a crucial role. The consumer selects from the product's most essential attributes. Acknowledging why a consumer selects a product based on its attributes enables us to comprehend why certain consumers chose or switched brands. Therefore, consumer decisions are dependent on optimizing the relevance of product attributes [42]. The product attribute is one of the most significant factors in determining the behavior of repeat purchasers and brand switchers [54]. In the initial phases of the product selection process, consumers mostly relied on attribute-based evaluations [46]. In contrast, other early research [26] demonstrates that the impact of product attribute characteristics on brand switching behavior is not statistically important. According to previous research by Ref. [55], only particular product attributes have a considerable impact on brand switching.

It has been determined that the FMCG product attributes of the brand are important for people to purchase it frequently and like it instead of switching brands [44,54,56,57]. Consumers tend to switch brands when there is no change in the attributes of their current products [58]. The majority of brands on the market provide regular discounts or specials across the majority of FMCG categories, and price-conscious customers will continue to switch brands when the brand is on sale [59]. According to an exploratory study of the specialist literature [60], consumers choose FMCG products based on price, quality, brand, advertisement, and packaging, among other essential product attributes. In contrast, a previous study by Ref. [26] revealed that product attributes do not have a considerable influence on brand switching behavior [55] found that specific product attributes impact brand switching significantly.

Variety seeking behavior is the tendency of consumers to “move from one brand to another” in order to enhance their pleasure by switching brands [61]. The preference for variety seeking behavior is positively connected with the chance of future brand switching [62]. Research by Ref. [63] revealed a significant direct influence of variety seeking on brand switching. As part of the post-purchase assessment process, consumers determine whether to continue purchasing the same brand or begin searching for and purchasing a new brand. Those who desire variety have a propensity to switch brands [64]. However, variety seeking is not always a factor in brand switching [65]. When there is a product promotion or discount, consumers are not driven by a need for product variety to switch brands.

It has also been demonstrated that variety seeking plays a key role in brand switching in FMCG category [66]. When there is no variety in the attributes or brands of the existing product, consumers switch brands [58]. Numerous consumer items have exhibited variation seeking behavior. Variety brings enjoyment to a life that might otherwise be monotonous. Variety-seeking consumers are characterized as those with a low chance of purchasing the same brand on their subsequent purchase occasion [67]. However, according to Ref. [65], variety-seeking does not always play a role in brand switching.

Dissatisfied consumers are motivated to investigate alternative choices available and may not purchase the product again or convince others to stop purchasing it. Consequently, consumer dissatisfaction is one of the most prevalent causes of brand switching [28]. Consumer dissatisfaction greatly influences brand switching [30]. Dissatisfied consumers are more likely to switch brands [27,63]. However, according to Ref. [68], dissatisfaction varies in relation to the likelihood of switching brands when product classes are distinct. A previous study by Ref. [17] revealed that consumer dissatisfaction does not always directly influence brand switching. It depends on cross-cultural variations.

It has been seen in FMCG that dissatisfaction with the previous brand is the primary cause leading to brand switching [44]. Customers that are dissatisfied are more inclined to switch brands [27]. Dissatisfied consumers are eager to investigate alternative product options and may not purchase the product again or convince others to stop purchasing it. Consequently, consumer dissatisfaction is one of the most prevalent causes of brand switching [28]. Consumer dissatisfaction greatly influences brand switching [30]. However, an earlier study by Ref. [17] found that consumer dissatisfaction does not always directly drive brand switching. It depends on the type of individual or group action.

This study used a partial least squares - structural equation model (PLS-SEM) to evaluate if the four factors mentioned above—bad prior experiences, bad product attributes, variety seeking, and consumer dissatisfaction—are the determining reasons for switching GUM product brands. In the meantime, multigroup analysis (MGA) approaches are employed to see whether there are differences in the brand-switching behavior of GUM customers living in rural compared to urban areas. To the author's knowledge, research on the switching of GUM product brands that integrate PLS-SEM and MGA has never been conducted. Typically, previous studies employed only single analytical approaches, such as confirmatory factor analysis (CFA) [26,69], multinomial logit [31], logistic regression [3], and structural equation modeling (SEM) [70].

### 2.2 Research Conceptual Framework and hypothesis formulation

Why is it necessary to include prior experience as a brand switching model variable? Based on the study [46], consumers' prior product experiences are a significant aspect of the consumer selection environment. Inexperienced customers need more time to evaluate product attributes before choosing a brand or switching brands. Typically, consumers evaluate purchases based on their previous experiences. Satisfaction is likely if the product meets the consumer's expectations, and conversely [71]. According to Refs. [[17], [18], [19]], bad previous purchasing experiences will drive consumers to switch brands. Consumers will continue to purchase the same products if their prior purchase experiences resulted in good outcomes; conversely, unsatisfactory outcomes result in brand switching [47,48].

Furthermore, product attributes play an important role in brand switching. If there are no products with adequate attributes, consumers may switch brands [42]. The consumers have preferences about the attributes or characteristics of products. Each product is a collection of attributes. The consumer picks among the product's most fundamental characteristics. When we understand why a consumer buys a product based on its attributes, we may discover why specific consumers selected or switched brands. Therefore, the customer's decision is centered on maximizing utility or the level of pleasure or dissatisfaction [42]. In the early stages of the product selection process, customers mainly utilized attribute-based assessments based on prior experience [46]. Product attribute is one of the most influential elements on the behavior of repeat buyers and brand switchers [54].

Moreover, brand switching behavior is typically preceded by dissatisfaction among consumers. Consumer dissatisfaction occurs when marketers' performance does not meet consumers' expectations. Consumers typically desire variety and are compelled to switch brands if dissatisfied with a previous purchase. Consumer dissatisfaction is correlated with brand switching [2,17,[25], [26], [27], [28], [29], [30]]. Dissatisfied consumers are eager to explore alternative options and may not make the purchase again or perhaps influence others to stop buying it. Consequently, consumer dissatisfaction is one of the most prevalent causes of brand switching [28].

In addition, variety seeking is also a major factor in brand switching. According to Ref. [72], variety seeking is described as “the need for a fresh or different stimulus.” It presents itself in numerous ways, including brand-switching behaviors and habits [73,74]. Consumers that desire product variety are more motivated to switch brands [31].

**Fig. 1:**
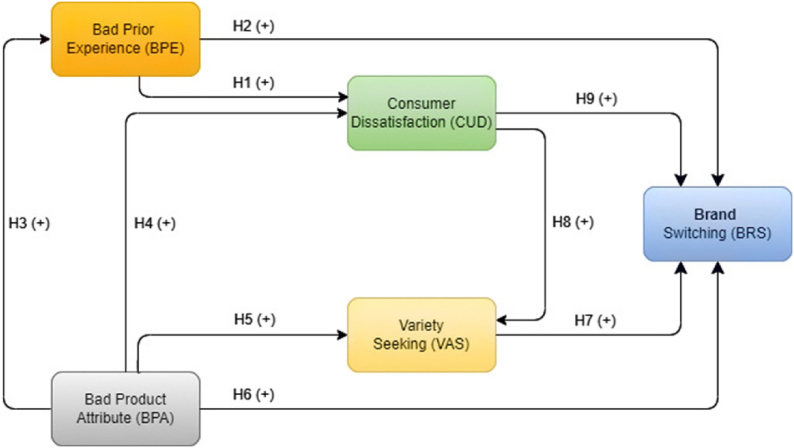
Research Conceptual Framework and hypothesis.

In consideration of previous research undertaken by various academics, we propose a brand switching model that includes four primary independent factors: bad prior experience, bad product attributes, variety seeking, and consumer dissatisfaction, as illustrated in Fig. 1.

In accordance with [17], the prior experience with the consumer of unsatisfactory consumption stimulates switching of product brands. Conversely, the good experiences consumers have had with previous purchases stimulates their proneness to satisfy and stay with the current brands [19]. Bad prior experience with previous purchases tends to encourage consumers to be dissatisfied and switch brands. A consumer may be satisfied or unsatisfied with many characteristics of the same product. Prior experience establishes the significance of experience in influencing satisfaction or dissatisfaction [18]. Typically, customers base their evaluations of purchases on their prior experiences. Dissatisfaction emerges if it does not satisfy consumer expectations [71].

H1 Bad Prior Experience has an association and positive with Consumer Dissatisfaction

Bad previous buying experiences will result in brand switching [[17], [18], [19]]. Customers will not continue to buy the same items if their previous purchases resulted in negative consequences; in other words, bad prior experiences lead to brand switching [47,48]. If a consumer has a negative experience with the present brand, they are more likely to switch to another brand, and likewise [75]. The primary factor that influences what individuals purchase today is what they previously experienced in purchasing, which could be understood as the impact of consumer inertia that causes consumers to switch brands [76].

H2 Bad Prior Experience has an association and positive with Brand Switching

Consumer interest in a product is heavily influenced by its attributes. While the product attributes are structured as an assembly of tangible attributes, such as instant packaging, color, and price. Intangible attributes include brand, and service. In contrast, customer satisfaction is the response of the consumer after acquiring a brand. It is considered that brand satisfaction results when consumer expectations and product advantages align [77]. Consumer dissatisfaction is the opposite condition, where the product benefits are lower than consumer expectations. Dissatisfied consumers are prone to looking for other product options and starting to seek the product variety that meets their needs and expectations.

In the early stages of the product selection process, customers mainly utilized product attribute-based assessments based on prior experience [46]. Internally, if consumers have a bad experience with a product's attributes, they will be more likely to switch brands [22]. As a consumer's ability to classify products based on their attributes grows, so does their prior product experience. A highly developed product attribute may convince experienced consumers that they do not need to do an information search prior to making a purchase decision [78].

H3 Poor Product Attribute has a significant and positive association with Bad Prior Experience

Typically, customers buy products based on attributes. Therefore, a thorough understanding of these characteristics enables us to comprehend why particular consumers choose or switch brands. Consumers will be dissatisfied if there are no items with suitable attributes [42]. Consumers may be satisfied with one attribute but dissatisfied with another. The product attribute-level approach gives a straightforward answer [79].

H4 Poor Product Attribute has a significant and positive association with Consumer Dissatisfaction

The consumers have preferences about the attributes or characteristics of products. Each product is a collection of attributes. The consumer picks among the product's most fundamental characteristics. If there are no items with sufficient attributes, consumers may seek product variety [42]. Product attributes that can meet customer demand are described in a taxonomy for seeking out product variations [80]. Certain product attributes make consumers have a higher variety seeking behavior [55].

H5 Poor Product Attribute has a significant and positive association with Variety Seeking Behavior

Repurchase decisions for the same brand are directly influenced by the product attribute [79]. A product's attributes are subject to consumer preferences. The consumer selects from the product's most essential attributes. In the absence of suitable items, consumers may switch brands [42]. A customer's inclination to switch brands isn't triggered by a single product attribute that doesn't meet their expectations [22]. Customers' decisions to choose or switch brands might be aided by a thorough understanding of the relevant product attributes [81]. Product attribute is one of the most influential elements on the behavior of repeat buyers and brand switchers [54].

H6 Poor Product Attribute has a significant and positive association with Brand Switching

Brand switching is significantly influenced by consumer dissatisfaction, which is moderated by variety-seeking [30]. Consumers decide whether to continue purchasing the same brand or start looking for and purchasing another brand as part of the post-purchase evaluation process. Variety seekers have a propensity to switch brands. Customer retention is negatively impacted by variety seeking behavior [64]. The preference for variation seeking behavior is negatively correlated with the likelihood that customers will retain the previous brand in the future [62]. Variable that has a strong direct influence on brand switching is variety seeking behavior [63]. In line with [61], variety seeking behavior is described as the tendency of customers to “switch from one brand to the other” in order to increase their value by switching brands.

H7 Variety Seeking Behavior has a significant and positive association with Brand Switching

The inclination of consumers to seek out other items or brands indicates that they are dissatisfied with what they have. Dissatisfied consumers are willing to seek product variety or investigate alternative options and may not purchase the goods again or discourage others from doing so [28]. Consumer dissatisfaction motivates the tendency to seek product variety [19]. When customers are dissatisfied with the product they are currently using, they will seek out other varieties [82].

H8 Consumer Dissatisfaction has a significant and positive association with Variety Seeking Behavior

A significant direct influence on brand switching is exerted by dissatisfaction [63]. Customers that are dissatisfied are more motivated to switch to a competitor. In contrast, brand loyalty is not decided by customer happiness. The link between satisfaction and loyalty is asymmetrical [27]. Dissatisfied consumers are motivated to investigate alternative product options, might not purchase the product again, and then convince others to stop purchasing it. Consequently, consumer dissatisfaction is among the most prevalent causes of brand switching [28]. In compliance with [30], brand switching is significantly influenced by consumer dissatisfaction.

H9 Consumer Dissatisfaction has a significant and positive association with Brand Switching

## 3 Method of research

### 3.1 Research location

The focus of the study is on how GUM customers from the middle and lower socioeconomic classes in Java, both rural and urban, switch brands. In the meantime, households with children between the ages of one and three who drink GUM and have switched brands of GUM products are the subject of the research.

Java, a prominent island in Indonesia, was chosen as a location for study for two reasons: its population and economics. Java Island is home to 56.1% of the total population of Indonesia (151,59 million people). Java group of provinces accounted for 58.75% of the Indonesian economy [83].

The research was conducted in two different provinces out of Java's six provinces. The first research location is East Java province, one of the biggest provinces in terms of population. The second location for study is Daerah Istimewa Yogyakarta (DIY), located in the center of the island of Java. DIY is one of the developed provinces on Java Island and is recognized as a “special region province” owing to its unique regulatory and administrative jurisdiction over its own territory.

### 3.2 Major instruments

Based on the measurement process, the variables are split into two parts: manifest variables (observable) and latent variables (unobservable). Generally, latent variables are defined as variables that cannot be measured directly but must go through their constituent indicators. Researchers are advised to use at least four variables, otherwise it will be hard to do an analysis. Difficulty in identification (unidentified) as well as estimation of error are not reliable if a model applies only two indicators to a latent variable [84].

The study has four independent variables, which are as follows: bad prior experience, bad product attribute, variety-seeking behavior, and consumer dissatisfaction. The dependent variable in the research is brand switching. Each construct will be translated into several indicators in the form of a guided questionnaire (see Table 1). There will be five further responses, each followed by a scale model.

**Table 1:** Constructs, indicators, code, reference, and loading factor.

Construct	Indicator (Code)	Reference	Loading Factor
Bad Prior Experiences (BPE)	• Previous disappointments with the old brand motivated me to switch to the new one. (BPE1)	[30] [44],	0.782
	• Previous experiences with defective (dented, lumpy, and odorous) products persuaded me to switch to the new one. (BPE2)	[13,31,66]	0.848
	• A history of products being unavailable at the store encouraged me to switch to the new brand. (BPE3)	[31,86]	0.658
Bad Product Attribute (BPA)	• I switched to the new brand because the old brand was too expensive for me. (BPA1)	[42,87,88]	0.526
	• The poorer quality of the previous product drove me to switch to the new brand. (BPA2)	[54,89]	0.436
	• Unattractive brand packaging influenced my decision to switch to a competitor ’s product. (BPA3).	[51,60]	0.684
	• The unfamiliar brand motivated me to switch to the new brand. (BPA4)	[90,91]	0.760
	• No claims regarding the advantages of probiotics affected my decision to switch brands (BPA5)	[19,92]	0.689
Consumer Dissatisfaction (CUD)	• I opted to switch brands since the previous brand's products were of poor quality. (CUD1)	[13,31,54]	0.737
	• The service of the brand was disappointing, therefore I switched to a different brand. (CUD2)	[93,94]	0.684
	• The undiscovered benefits of probiotics prompted me to switch to a different brand. (CUD3)	[44,92]	0.821
Variety Seeking (VAS)	• I determined to switch to the new brand since the old company's advertising was lacking. (VAS1)	[65]	0.733
	• I identified a brand with a lower pricing and decided to switch to it. (VAS2)	[89]	0.720
	• I discovered that the previous brand was no longer available at the shop and chose to switch to the new one. (VAS3)	[86]	0.577
Brand Switching (BRS)	• I was dissatisfied with the previous brand and chose to switch to the new one. (BRS1)	[27]	0.670
	• The old brand's inferior quality prompted me to switch to the new brand. (BRS2)	[35,95]	0.784
	• The services provided by the brand were insufficient, consequently I switched brands (BRS3)	[75,90]	0.788
	• I became bored of the previous brand and decided to switch to the new one. (BRS4)	[96,97]	0.566

The scale model that is often used is the Likert scale. The determination of the response item in the form of a score in general is five, which is 1 for severely disagree, 2 for disagree, 3 for neutral, 4 for agree, and 5 for strongly agree. Furthermore, the selection criteria are based on attempts to generate the most precise data [84]. Likert scale, with five points classified as the interval scale [85].

### 3.3 Method of sampling and data collection

The respondents have been divided among two provinces of Java, namely East Java province and Daerah Istimewa Yogyakarta (DIY) province. The data was gathered from two regencies, Bantul (DIY) and Malang (East Java), which represented rural areas, and two municipalities, Yogyakarta (DIY) and Malang (East Java), which represented urban areas. The data was collected through face-to-face interviews guided by a questionnaire survey.

Before participating in and completing this survey, all respondents provided their consent. The study was conducted between May and September 2021. This study's participants were selected using a technique known as purposive sampling and were chosen according to the following inclusive criteria: 1) Respondents are members of integrated health service posts/IHSP (*Posyandu*); 2) respondents are GUM purchasers; 3) the respondent has children aged one to three who consume GUM; and 4) they have switched brands at least once.

Initially, 1493 Posyandu member records were gathered from leaders of the *Posyandu* cadre. There were 1029 GUM consumers, 584 of whom had switched brands at least once, and 419 who were willing and able to be interviewed. The 419 respondents consisted of 105 GUM consumers from the Bantul Regency, 111 from the Malang Regency, 101 from the Yogyakarta Municipality, and 102 from the Malang Municipality. The number of participants in our study meets the minimal threshold for PLS-SEM application. PLS-SEM requires a sample size of at least ten times the number of arrowheads pointing toward our latent variable (9 orientations), which equals 90 respondents at minimum [98].

Elements of the survey were constructed based on a review of prior studies on brand switching behavior. Table 1 provides a full overview of the research concept, indicators, and references. The objective of the interviews was to identify influential perspectives and attitudes around brand switching. A self-designed questionnaire was employed in this study.

In this study, a structured, two-part questionnaire was utilized. The first portion included socio-demographic information about the respondents. Included are the respondent's sociodemographic factors, such as age, education, occupational status, the status of consuming GUM products, the number of glasses consumed per day, the age of young children who consume GUM, the brand name of GUM that was consumed, the name and number of brands ever purchased, and monthly household expenditure.

The second segment comprised a series of surveys designed to assess the structural aspects of brand switching among consumers, including 18 indicators from five variables: (1) three BPE indicators, (2) five BPA indicators, (3) three CUD indicators, (4) three VAS indicators, and (5) four BRS indicators (Table 1). Each indicator reflected the relevant BPE, BPA, CUD, VAS, and BRS constructs.

### 3.4 Data analysis

The study examined the degree of brand switching for GUM across all brands, rather than for each specific brand. The study computes the proportion of GUM users who have switched brands relative to the total number of GUM consumers properly observed in such a given region. This study also assesses the opposite case, namely GUM users who have never switched brands and have always purchased the same brand, such that they become brand-loyal. The overall number of GUM consumers is the sum of consumers who have switched brands and loyal consumers. All of these data were gathered through the completion of questionnaires by respondents. The brand switching level is calculated based on the brand loyalty level, where the calculation refers to Ref. [10].

When the number of brand switches declines between two purchases, this indicates brand loyalty. Low brand loyalty results in frequent brand switching [70,99]. Brand switching is the inverse of brand loyalty, and the higher rate of brand switching indicates a lower level of brand loyalty. If brand loyalty can be quantified, it will be simple to determine how frequently consumers switch brands. According to Ref. [10], brand loyalty may be quantified by their persistent brand repurchases, which is called the behavioral perspective (stochastic). Determining brand loyalty is the proportion of total purchases attributed to the favorite brand. This metric is based on the proportion of purchases with hard core criteria (the single most frequently purchased brand). Consumers are considered loyal if they purchase over 75% of their preferred brand In other words, a brand switching rate of greater than 25% is regarded as significant, and consumers are considered unloyal.

This study furthermore performed partial least square (PLS) analysis as part of structural equation modeling (SEM) to evaluate the research model and its predictive value. PLS modeling is a prediction method based on variance that optimizes variance explanations by concentrating on endogenous model constructs [98], which is appropriate for exploratory study objectives [100]. In line with [101] PLS-SEM is advantageous for issues involving limited sample sizes and non-normal data. In consequence, PLS-SEM is a preferable alternative to covariance-based SEM since it allows the construction and estimation of a specific model without taking into consideration highly restricted limitations [101,102].

PLS-SEM and confirmatory factor analysis (CFA) employing WarpPLS software (version 7.0) were used to analyze the data as well as evaluate the hypotheses of this research. To address our hypothesis, we shall take into consideration two measurement phases. The examination of the outer model starts with the measurement of the indicator's reliability, convergent validity, internal consistency, and discriminant validity. The overall model fit was then assessed using the Tenenhaus GoF criterion [103,104].

Interpretation of hypothesis tests could only be carried out when the quality indices and the goodness of fit (GoF) of the correlations amongst latent variables of the research model were “Fit” or “Acceptable”. Table 2 proves that the proposed model is good and that all of the parameters and the WarpPLS quality index are met.

**Table 2:** Quality indices and goodness of fit model.

Parameters	Result	Norm	Remarks
Average Path Coefficient/APC	0.211; p < 0.001	p < 0.05	Fit
Average R^2^/ARS	0.149; p < 0.001	p < 0.05	Fit
Average Adjusted R^2^/AARS	0.144; p < 0.001	p < 0.05	Fit
Average Block VIF/AVIF	1.084	accept if ≤ 5; ideally ≤3.3	Fit
Average Full Collinearity VIF/AFVIF	1.235	accept if ≤ 5; ideally ≤3.3	Fit
Tenenhaus GoF/GoF	0.309	small ≥0.1; medium ≥0.25; large ≥0.36	Fit
Sympson's Paradox Ratio/SPR	1.000	accept if ≥ 0.7; ideally = 1	Fit
R [2] Contribution Ratio/RSCR	1.000	accept if ≥ 0.9; ideally = 1	Fit
Statistical Suppression Ratio/SSR	1.000	accept if ≥ 0.7	Acceptable
NonLinear Bivariate Causality Direction Ratio/NLBCDR	1.000	accept if ≥ 0.7	Acceptable

In the SEM model of the research, consumer brand switching is classified as an endogenous latent variable, whereas bad prior experience, bad product attributes, variety seeking, and consumer dissatisfaction are considered exogenous latent variables. For measuring the latent variable, multi-item scales were utilized. The SEM consists of both measurement models and structural models. This model describes the relationship between observable and latent variables. While endogenous and exogenous latent variables are interrelated in the structural model. Three matrix equations compose the structural equation model, as follows:

(1) β=Aβ+Bλ+ζ

(2) X=Λxλ+v

(3) Y=Λyβ+ε

The structural model is represented by Equation (1), where β represents the endogenous latent variable, λ represents the exogenous latent variable. The relationship between the endogenous and exogenous latent variables is connected via the coefficient matrices A and B, and ζ represents the error vector. Equations (2), (3) illustrate the measurement models. X is the observed exogenous latent variable, while Y is the observed endogenous latent variable. Λx is the matrix of correlation coefficients between both the exogenous variable and its observed variable, whereas Λy is the matrix of correlation coefficients between the endogenous variable and its observed variable.

In addition, research employs multigroup analysis (MGA) to compare brand switching behavior between rural and urban areas. It needs to be done manually when conducting comparative analysis or multi-group analysis between rural and urban groups. The data required is the value of the regression weights (path coefficients) as well as the standard errors for the path coefficients. Both are available in the PLS graph, as is the number of samples in each group. Then compute the *t*-test for path differences among groups. Fundamentally, using t-tests, we must run bootstrap re-samplings for each group and treat the standard error as evaluated from each re-sampling in the sense of parametric error [105].

**Fig. 2:**
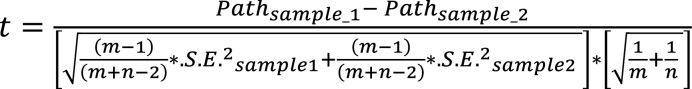
Formula *t*-test for Multi Group Analysis.➢ Path _sample_1_: Coefficient of Path (Group 1)➢ Path _sample_2_: Coefficient of Path (Group 2)➢ m: Respondent Number of Group 1➢ n: Respondent Number of Group 2➢ S.E._sample1_: Standard Error of Inner Model of Group 1➢ S.E._sample2_: Standard Error of Inner Model of Group 2 Notes:

In this study, the behavior of brand-switching in Java's rural and urban groups was compared using a multigroup analysis referred to as the following formula [105] (Fig. 2):

$$t=\frac{{Path}_{sample\_1}-{Path}_{sample\_2}}{\left[\sqrt{\frac{\left(m-1\right)}{\left(m+n-2\right)}*.\;{S.E.}_{sample1}^{2}+\frac{\left(m-1\right)}{\left(m+n-2\right)}*.\;{S.E.}_{sample2}^{2}}\right]*\left[\sqrt{\frac{1}{m}+\frac{1}{n}}\right]}$$

## 4 Research result and discussion

### 4.1 Descriptive statistical

Empirical data was obtained from the profiles and characters of the respondents listed in the questionnaire. The level of brand switching is the ratio between the number of respondents who drink GUM and have switched brands, divided by the total number of respondents who drink GUM. This data will be compiled and then adequately explained according to the research objectives.

According to Table 3, the average household expenditure is IDR 2.0 million per month, which is still within the range of expenditures for the middle and lower socioeconomic classes, which is less than IDR 5 Mio [36]. The vast majority of respondents (99.3%) were female. Most of them purely act as housewives (75.4%), and several of them are employed (26.9%). Around 73.1% of respondents have completed senior high school or a lower level of education, while the remaining 26.9% have earned a diploma or a higher education.

**Table 3:** Number, gender, monthly household expenditure, jobs, and education of respondent.

Number	Gender		Monthly Household Expenditure (Mio IDR)			Job/Occupation			Education	
	Female	Male	min	max	avg	Housewife	Private	Civil Servant	Up to Senior High School	Diploma & above
419	99.3%	0.7%	0.5	4.8	2.0	75.4%	22.7%	1.9%	73.1%	26.9%

To determine the level of brand switching for GUM products, the following information is required: the total number of Posyandu, the number of active members present, the number of balita who drink milk formula, and the member of Posyandu who has changed the brand of their GUM products. The data is collected from some coordinator member of Posyandu who has been appointed by the head of the public health office (Kepala Dinas Kesehatan Kota/Kabupaten). There are a total of 21 sub-districts out of the total area studied. The following table illustrates the distribution of sub-districts: Malang municipality has five sub-districts, Malang Regency has four sub-districts, Yogyakarta municipality has four sub-districts, and Bantul Regency has eight sub-districts.

The following criteria were used to determine *Posyandu*: the number of children aged one to three years old (*batita*), the degree of attendance of Posyandu's members, and their willingness to participate in interviews throughout the research period. Research reveals that among the 1029 *batita* who consumed GUM, 584 *batita* has switched brands, which equals a 57% switching rate and is considerably high (Table 4). It means that one out of every two consumers has changed their brand after buying a GUM product. The results of the research confirm the earlier findings that, with the lowering level of household income, there was a significant tendency toward increased brand switching prevalence [106].

**Table 4:** Level of switching, milk consumption, active member, and brand consumed.

Brand Switching Level	Average of Milk consumption (glass/day)	Active Member of Posyandu	Brand Consumed	
			Affordable &Mainstream	Premium
**57%**	3.5	87%	65.7%	34.3%

In both rural and urban areas, consumers in the lower and middle socioeconomic classes who buy GUM products tend to frequently switch brands. They purchase GUM in small pack sizes to save money and tend to switch to a different brand whenever possible. Finding products that are out of stock in stores is a truly bad experience for consumers, and as a result, they change brands to ones that are available and ready in stores. In addition, diarrhea and constipation are two of the most frequently reported sources of consumer dissatisfaction. For this reason, consumers are prompted to switch from old brands to new brands.

The study revealed that milk consumption is 3.5 glasses per day on average. It is an excellent indicator of daily milk consumption due to the fact that the real quantity is above the minimum suggested consumption, which is 300 ml, or two glasses per day [107]. The rate of attendance of Posyandu members is exceptionally high, at 87%. A Posyandu is classified as having a high attendance rate when more than 85% of members are active, according to standards set by health agency. In rural and urban areas, 65.7% of consumers purchase affordable and mainstream brands such as Dancow, Batita (Nestlé), Flag (Frisian Flag), Vidoran Xmart (Scan Tempo), and SGM (Danone).

This study discovered a phenomenon as well as a surprising trend in consumer behavior: 34.3% of consumers in the middle and lower socioeconomic classes consumed the premium GUM brand. Typically, affluent and elite consumers choose to purchase premium brands. Here are some examples of premium brands: Pediasure from PT Abbott which is an imported product, Chil Kid from PT Kalbe–Morinaga, Enfagrow from PT Mead Jhonson Nutrition, and Nutrilon and Bebelac products from PT Nutricia, and PT Danone Indonesia. This phenomenon happened because consumers perceived that premium brands were associated with higher quality products, and they wanted to get the best product quality for their children, even though they had to be willing to pay a higher price. In certain cases, consumers are not price-sensitive [90].

### 4.2 Inferential statistical result

#### 4.2.1 Test of validity for every indicator (Covergent validity and discriminant validity)

Convergent validity is defined as a set of indicators that both represent and underlie a latent variable. The coefficient of correlation between the scores of the reflective indicator and the scores of the latent variable is reflected in the value of the loading factor. An indicator is called meeting convergent validity when the loading factor ≥0.5 or 0.6 ^84^, even if the loading factor ≥0.30 [108]. All indicators comply with convergent validity, as shown in Table 5.

**Table 5:** Loading factor and cross loadings.

	BPE	BPA	CUD	VAS	BRS	Type	SE	*p-value*	Remarks
BPE1	0.782	0.043	−0.084	−0.133	0.039	Reflective	0.044	<0.001	Valid
BPE2	0.848	0.008	0.061	−0.128	0.059	Reflective	0.044	<0.001	Valid
BPE3	0.658	−0.061	0.021	0.323	−0.122	Reflective	0.045	<0.001	Valid
BPA1	−0.057	0.526	−0.104	0.376	−0.001	Reflective	0.046	<0.001	Valid
BPA2	0.040	0.436	0.004	−0.300	0.043	Reflective	0.046	<0.001	Valid
BPA3	0.153	0.684	−0.163	0.066	0.090	Reflective	0.045	<0.001	Valid
BPA4	−0.005	0.760	0.004	−0.078	−0.015	Reflective	0.044	<0.001	Valid
BPA5	−0.128	0.689	0.233	−0.076	−0.099	Reflective	0.045	<0.001	Valid
CUD1	−0.150	0.079	0.737	−0.043	−0.050	Reflective	0.044	<0.001	Valid
CUD2	0.145	−0.128	0.684	−0.057	0.099	Reflective	0.045	<0.001	Valid
CUD3	0.014	0.036	0.821	0.086	−0.038	Reflective	0.044	<0.001	Valid
VAS1	−0.074	0.058	−0.028	0.733	0.017	Reflective	0.044	<0.001	Valid
VAS2	−0.046	0.032	−0.037	0.720	−0.007	Reflective	0.044	<0.001	Valid
VAS3	0.151	−0.113	0.083	0.577	−0.012	Reflective	0.045	<0.001	Valid
BRS1	0.001	−0.083	0.331	−0.161	0.670	Reflective	0.045	<0.001	Valid
BRS2	0.071	0.041	−0.213	−0.032	0.784	Reflective	0.044	<0.001	Valid
BRS3	0.025	−0.006	−0.027	−0.033	0.788	Reflective	0.044	<0.001	Valid
BRS4	−0.134	0.051	−0.058	0.280	0.566	Reflective	0.045	<0.001	Valid

Note(s): Cross loadings (oblique rotated) while loadings are unrotated. Standard Errors and *p-values* are only for loadings and for reflective indicators *p-values* < 0.05 are preferable.

Comparing the difference between cross loading and loading values is a simple method for determining the values of discriminant validity. An indicator of one latent variable is said to be discriminantly valid if its loading value (cross loading) is smaller than the loading value of every other indicator on the relevant variable [84]. Table 5 shows that each indicator has a loading value (cross loading) smaller than the loading factor for every indicator on relevant variables, and it is identified as having discriminant validity.

#### 4.2.2 Reliability and validity test for overall variable

As part of the examination of the outer model, it is important to analyze the reliability of the entire questionnaire for all variables. This study makes use of reliability test parameters, namely the composite reliability coefficient (acceptable value > 0.7) and Cronbach's alpha coefficient (desired value > 0.6). At the same time, the overall validity of the questionnaire was measured by the average variance extracted/AVE (target value > 0.5) [84].

The study's findings (Table 6) revealed that all five latent variables—Bad Prior Experience (BPS), Bad Product Attribute (BPA), Customer Dissatisfaction (CUD), Variety Seeking Behavior (VAS), and Brand Switching (BRS)—complied with the parameters of the questionnaire's reliability and validity test.

**Table 6:** Reliability and validity test for overall questionnaire.

No	Variable	Coefficient of Reliability		Convergent Validity	Remarks
		Composite Reliability	Cronbach's alpha	Average Variance Extracted	
1	BPE	0.809	0.644	0.588	Valid
2	BPA	0.795	0.612	0.566	Valid
3	CUD	0.793	0.607	0.562	Valid
4	VAS	1.000	1.000	1.000	Valid
5	BRS	0.798	0.661	0.501	Valid

The output correlations between latent variables and the square roots of AVEs were also utilized to examine the questionnaire's divergent validity in a different ways. According to the findings of the research (Table 7), each value in the diagonal column of the questionnaire has a correlation that is greater than the correlation between the latent variables in the same column, either above or below it. This indicates that the questionnaire has valid divergent data and is therefore valid [84].

**Table 7:** Divergent validity test for overall questionnaire.

	BPE	PPA	CD	VS	BS
BPE	**0.767**	0.139	0.228	0.123	0.398
PPA	0.139	**0.752**	0.357	0.189	0.258
CD	0.228	0.357	**0.749**	0.139	0.407
VS	0.123	0.189	0.139	**1.000**	0.216
BS	0.398	0.258	0.407	0.216	**0.708**

Note: Square roots of average variances extracted (AVEs) shown on diagonal.

#### 4.2.3 Hypothesis result

**Fig. 3:**
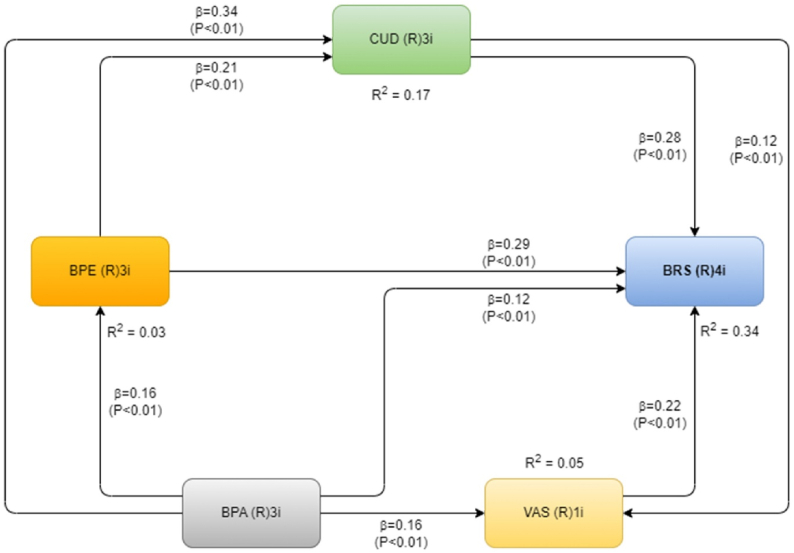
SEM analysis result.

Table 8 shows the results of testing the hypothesis. The results are given as standardized path coefficients. It emphasizes the significance of relationships between latent variables. Fig. 3 depicts the findings of our research.

**Table 8:** The structural model and hypothesis testing result.

Hypothesis	Correlation of Variables (independent → dependent)			Path coefficient	*p-value*	Remark
H1	BPE	→	CUD	0.211	<0.001***	Significant
H2	BPE	→	BRS	0.286	<0.001***	Significant
H3	BPA	→	BPE	0.162	<0.001***	Significant
H4	BPA	→	CUD	0.338	<0.001***	Significant
H5	BPA	→	VAS	0.158	<0.001***	Significant
H6	BPA	→	BRS	0.125	0.005***	Significant
H7	VAS	→	BRS	0.224	<0.001***	Significant
H8	CUD	→	VAS	0.117	0.008***	Significant
H9	CUD	→	BRS	0.277	<0.001***	Significant

Note(s): ***Significant at α 1%; ** Significant at α 5%; * Significant at α 10%.

BPE = Bad Prior Experience; BPA = Bad Product Attribute; CUD = Consumer Dissatisfaction; VAS = Variety Seeking; BRS = Brand Switching.

According to the findings, all the variables, which were bad prior experience (BPE), bad product attribute (BPA), variety seeking (VAS), and consumer dissatisfaction (CUD) among GUM consumers have a positive and significant influence on brand switching (BRS). As a consequence of this, we have decided to accept H2, H6, H7, and H9. With a path coefficient value of 0.286, BPE had the greatest direct impact on brand switching. CUD, VAS, and BPA came in second through fourth, with path coefficient values of 0.277, 0.224, and 0.125, respectively.

In addition, BPA has a significant statistical and positively influential effect on BPE, CUD, and VAS, with respective path coefficient values of 0.162, 0.338, and 0.158. In light of the finding, this means that the H3, H4, and H5 hypotheses are accepted. Compared to BPE and VAS, BPA has a path value of 0.338, which means it has a bigger direct effect on the level of customer dissatisfaction (CUD).

Furthermore, BPE has a statistically significant and positively influential effect on CUD, and has a path coefficient of 0.211. CUD also has a statistically significant and favorable influence on VAS, and has a path coefficient of 0.117. Consequently, we must accept H1 and H8.

The path analysis showed that among the constructs of bad prior experience (BPE), bad product attribute (BPA), variety seeking (VAS), and customer dissatisfaction (CUD), BPE is the most influential factor on brand switching among GUM consumers, with a path coefficient value of 0.286. This indicates that a bad prior experience is an extremely influential determinant in determining whether GUM consumers switch brands.

This finding is consistent with the theory of prior purchasing experiences [[17], [18], [19]]. This revealed that the construct of bad prior experience is one of the dominant factors that determine brand switching. According to Refs. [47,48] consumers will not continue to buy the same items if their previous purchases resulted in negative consequences; in other words, bad prior experiences lead to brand switching.

According to a number of past studies that have drawn the same conclusion, BPE has had the most significant role in influencing GUM consumers' decisions to switch brands. When a consumer has a bad experience with the present brand, they are more likely to switch to a different brand, and likewise [75]. According to Ref. [76], prior purchase experience has a considerable impact on present purchasing.

Furthermore, compared to BPE and VAS, BPA has a path value of 0.338, which means it has a bigger direct effect on the level of customer dissatisfaction (CUD). This implies that bad product attribute is very important variable in affecting GUM consumer dissatisfaction. This finding is consistent with previous research on the influence of product attribute [42], which found that the construct of a bad product attribute is the most influential factor in influencing consumer dissatisfaction.

According to Refs. [42,79], consumers buy products based on attributes; therefore, a thorough understanding of these characteristics enables us to comprehend why particular consumers choose or switch brands. Consumers will be dissatisfied if there are no items with suitable attributes. Consumers may be satisfied with one attribute but dissatisfied with another. The product attribute-level approach gives a straightforward answer.

Moreover, bad prior experience (BPE) has a significant statistical and positively influential effect on consumer dissatisfaction (CUD). This result is in line with previous studies that revealed that the prior experience of the consumer with unsatisfactory consumption stimulates switching of product brands [17]. The bad experiences consumers have had in previous purchases stimulate consumers' proneness to dissatisfy and switch the current brands [19]. Bad prior experience with previous purchases tends to encourage consumers to be dissatisfied and switch brands. A consumer may be satisfied or unsatisfied with many characteristics of the same product. Prior experience establishes the significance of experience in influencing satisfaction or dissatisfaction [18]. Typically, customers base their evaluations of purchases on their prior experiences. Dissatisfaction emerges if it does not satisfy consumer expectations [71].

In addition, consumer dissatisfaction (CUD) has a statistically significant and positively influential effect on variety seeking (VAS). This result implies that dissatisfied consumers tend to seek other brands of products. According to Ref. [82], when customers are dissatisfied with the product they are currently using, they will seek out other varieties. Meanwhile [19], revealed that consumer dissatisfaction drives the desire for product variety. On the other side, the tendency of consumers to seek out other products or brands indicates dissatisfaction with their current choices. Dissatisfied consumers are willing to seek product variety or investigate alternative options and may not purchase the goods again or discourage others from doing so [28].

The coefficient of R-squared for brand switching (BRS) in this research is 0.338 (Table 9). This score explains that the influence of the independent variables bad prior experience (BPE), product attributes (BPA), customer dissatisfaction (CUD), and variety seeking behavior (VAS) on the dependent variable brand switching (BRS) is 33.8%. Based on [109], R^2^ with value of 0.33, it is categorized as moderate. The remaining 66.2% is explained by variables or factors beyond the scope of the study, such as demographic variables such as gender, age, education level, and occupation, in addition to background and cultural influences. Meanwhile, the remaining 66.2% is defined by other variables or other factors outside the study, for instance, factors of demographics such as age, education level, gender, and occupation, and other matters related to background and culture.

**Table 9:** R^2^ coefficients.

Adjusted R^2^ coefficients				
BPE	BPA	CUD	VAS	**BRS**
0.024		0.170	0.045	**0.338**

Empirically, some consumers from this middle and lower segment even purchase premium or expensive products. It means that a good price (cheap) does not always encourage consumers to buy the brand when consumers face a bad prior experience indicator, such as a defective product (dented, lumpy, or odorous).

#### 4.2.4 Multi-group analysis (MGA)

PLS-SEM provides several benefits and strengths, such as chain of effects or mediation, and allows for complex models that include unobserved or latent variables. In addition, it is possible to perform comparisons on groups of more complex relationships. Analysis of multigroups such as region, country, gender, and age can also be analyzed by PLS [105]. The differences in structure across the demographic groupings, like rural or urban areas, could be examined and analyzed using WarpPLS.

It needs to be done manually when conducting comparative analysis or multi-group analysis between rural and urban groups. The data required are the value of the regression weight (path coefficients) and the standard errors for the path coefficients. Both are available in the PLS Graph, as is the number of samples in each group. Then compute the *t*-test for path differences among groups. Fundamentally, via t-tests, we need to run re-samplings of bootstrap for the diverse groups as well as treat the standard error as evaluated from every resampling in the sense of parametric [105].

The research found (Table 10) that there is no difference in the behavior of consumers of the GUM product brand when switching products in the middle and lower socioeconomic classes between rural and urban areas in Java. The finding is aligned with earlier research [110], which found that brand switching is not mostly influenced by personal dimensions such as residence or domicile. This situation will be beneficial for marketers of GUM products, as the identical marketing strategy approach may be used in both rural and urban areas in Java. Hence, it does not necessitate an alternative strategy, which is typically more expensive. However, this discovery contrasts the findings of previous research, which revealed that urban consumers are more likely to prefer the most popular brands than rural ones [39], [40]. This study shows that the rural or urban location of GUM users from the same socioeconomic class has little influence on their brand preference or propensity to switch brands.

**Table 10:** Result of Significancy Level (*p value*) of Multi Group Analysis (MGA).

No	Variables correlation (independent → dependent)	Rural vs Rural	Urban vs Urban		Rural vs Urban	Remarks
1	BPE → BRS	0.320	0.276	0.073		NOT significant
2	BPA → BRS	0.864	0.176	0.177		NOT significant
3	CUD → BRS	0.363	0.169	0.739		NOT significant
4	VAS → BRS	0.281	0.356	0.383		NOT significant

Note(s): * *Significant at α 5%*.

### 4.3 Theoretical implications

The research examines the significance of bad prior experience, bad product attributes, variety seeking, and consumer dissatisfaction, on brand switching by GUM consumers. It was discovered that each of these factors had a different degree of influence on switching brands. With a path value of 0.286, bad prior experience is the greatest factor that influences brand switching of GUM consumers. It implies that consumers with bad prior experiences tend to switch brands. Prior experiences with defective (dented, lumpy, and odorous) items prompted consumers to switch to the new brand, which is the most prominent indicator of the theory of bad prior experiences.

This outcome validates the theory's applicability in prior studies by Refs. [17,19]. It has been discovered that a bad customer's prior experience is the motivating factor for switching brands. Because this study used GUM consumers as the research subjects, which is different from all previous studies, the findings strengthen the prior study's theory.

The findings are also consistent with the earlier research, which showed that a bad previous purchasing experience was a driving factor in switching brands [18]. According to Ref. [76], prior purchasing experiences have a significant influence on present purchases. Customers will not continue to buy the same items if their previous purchases resulted in negative consequences, which is why bad prior experiences lead to brand switching [47,48]. When a customer had a bad experience with the brand they were currently using, there was a greater likelihood that they would switch to a different brand [75].

The research indicated no difference between rural and urban locations in regards to the factors that influence GUM consumers' brand switching. This conclusion contradicts findings from a prior study performed by Refs. [39,40] revealed that urban consumers are more inclined than rural consumers to choose the most popular brands. This study demonstrates that the rural or urban location of GUM consumers from the same socioeconomic class has no effect on their brand preference or willingness to switch brands.

### 4.4 Practical implications

This study investigates the impact of bad prior experiences, bad product attributes, variety seeking, and consumer dissatisfaction, on GUM customers to switch brands. Bad prior experience is the greatest factor influencing GUM customer brand switching. It implies that consumers with a propensity for bad brand experiences are inclined to switch brands. The strongest indicator of the hypothesis of bad prior experience is the fact that consumers switched to a new brand after experiencing defective (dented, lumpy, and odorous) products in the past.

The research findings offer significant insights into elements of the milk industry, specifically GUM producers. Through their business or commercial units, particularly the marketing division, as well as technical units, in particular the division of product research and development, they have to be more focused on the improvement of product quality, mainly issues related to consumers who experience defective products such as dented, lumpy, and odorous. The quality management system has to guarantee that the GUM product is free from dented packaging, lumpy product, and odorous product. The best quality must be maintained at a superior level along the whole chain of production, from manufacturing until the product is consumed by consumers.

Defective products are the primary reason for bad purchase experiences. As a consequence, customers will be more inclined to explore other brands, as there will be a rise in acceptability of other brands. To be a market leader, managers must focus on offering the best quality products to give the best purchase experience to consumers [111].

This study demonstrated that there is no difference in the brand-switching behavior of rural and urban consumers; hence, the marketing strategy approach for both types of locations may be carried out in the same manner to increase efficiency.

### 4.5 Limitations and future research

Similarly to any assessment, this study has limitations. The limitation is a value of 33.8%for the coefficient of determination (R2), which is classified as moderate according to Ref. [109]. This is because the type of product used in this research is a highly segmented one, namely the GUM product. The reflecting indicators utilized in each construct were designed and created from scratch. Beyond the scope of the study, characteristics like demographic factors (age, education level, gender, and employment) as well as background and cultural influences account for the remaining 66.2% of factors influencing brand switching among GUM customers. These aspects of the study might be the subject of further research.

## 5 Conclusion

According to the study, GUM consumers in Java's middle and lower socioeconomic classes switched brands at a rate of 57%. It was considered high and created significant fluctuations in market share across GUM manufacturers. The most important factor influencing the brand switching behavior of GUM consumers in Java's middle and lower socioeconomic classes is bad prior experiences, followed by variety seeking, bad product attributes, and consumer dissatisfaction. A defective product is the most reflective indicator of a bad prior experience. This study revealed that there is no difference in brand switching behavior between rural and urban consumers in Java from the middle and lower socioeconomic classes. In the process of brand switching, the distinction between rural and urban customers is not based on their geographical area but on their socioeconomic status.

## Author contribution statement

Sunardi: Completed the entire field work, evaluated and analyzed the data and prepared the figures; Composed the paper. Jangkung Handoyo Mulyo: Interpreted the data; Providing powerful insights into the whole preparation and writing the manuscript; proofreading the manuscript; Drafted the paper. Irham: Providing advice and proofreading the manuscript. Jamhari: Making recommendations and proofreading the manuscript.

## Funding statement

This study received no specific support from public, corporate, or non-profit public entities.

## Data availability statement

Upon certain request would the data be publicly disclosed.

## Declaration of competing interest

This research is the final version and more comprehensive version of the preliminary research (https://iopscience.iop.org/article/10.1088/1755-1315/905/1/012087/pdf) in Malang City by the same authors. This research also used Multi Group Analysis (MGA) in addition to Structural Equation Modeling (SEM), and has a wider scope area by covering two provinces and four districts or cities. Therefore, comparisons can be made between urban and rural areas both in different provinces and within provinces.
